# A Review of Neural Network-Based Image Noise Processing Methods

**DOI:** 10.3390/s25196088

**Published:** 2025-10-02

**Authors:** Anton A. Volkov, Alexander V. Kozlov, Pavel A. Cheremkhin, Dmitry A. Rymov, Anna V. Shifrina, Rostislav S. Starikov, Vsevolod A. Nebavskiy, Elizaveta K. Petrova, Evgenii Yu. Zlokazov, Vladislav G. Rodin

**Affiliations:** Laser Physics Department, Institute for Laser and Plasma Technologies, National Research Nuclear University MEPhI (Moscow Engineering Physics Institute), Kashirskoe Shosse 31, 115409 Moscow, Russia

**Keywords:** camera noise, denoising, noise estimation, convolutional neural networks, generative adversarial networks, image noise, deep learning, camera source identification, photo-response non-uniformity, synthetic image

## Abstract

This review explores the current landscape of neural network-based methods for digital image noise processing. Digital cameras have become ubiquitous in fields like forensics and medical diagnostics, and image noise remains a critical factor for ensuring image quality. Traditional noise suppression techniques are often limited by extensive parameter selection and inefficient handling of complex data. In contrast, neural networks, particularly convolutional neural networks, autoencoders, and generative adversarial networks, have shown significant promise for noise estimation, suppression, and analysis. These networks can handle complex noise patterns, leverage context-specific data, and adapt to evolving conditions with minimal manual intervention. This paper describes the basics of camera and image noise components and existing techniques for their evaluation. Main neural network-based methods for noise estimation are briefly presented. This paper discusses neural network application for noise suppression, classification, image source identification, and the extraction of unique camera fingerprints through photo response non-uniformity. Additionally, it highlights the challenges of generating reliable training datasets and separating image noise from photosensor noise, which remains a fundamental issue.

## 1. Introduction

With the rapid development of digital and computer technologies, digital cameras have become an indispensable tool for various applications [1]. The precision of recorded images can be critical in fields such as forensics [2], copyright protection [3], and medical diagnostics [4]. One of the most important factors affecting the quality of the recorded images is digital camera noise [5]. Despite the fact that this noise often cannot be perceived by the human eye, it can significantly degrade the quality of images, and thus, the accuracy of their analysis. Therefore, noise analysis, evaluation, and suppression are crucial tasks in digital image processing.

Traditional methods for estimating and suppressing noise in images have a number of limitations, such as the need for parameter selection and low efficiency when dealing with large amounts of data or high noise levels [6]. With the advancements in neural network (NN) technology, NN-based methods are emerging that can be more efficient than traditional approaches. Neural networks, including convolutional neural networks (CNN), are already showing good results in extracting noise parameters from images, as well as in extracting unique noise characteristics such as pixel’s photo response non-uniformity (PRNU), which can be important, for example, for image source identification tasks [2].

The adaptability of NN processing allows us to take into account the peculiarities of signal-to-noise ratios in different images, handle heterogeneous data, and continuously improve the NN by adding new training samples. This is especially important as camera resolutions increase and signal distortion models become more complex, including both electronic noise and compression artifacts. In addition, NN algorithms can more effectively take into account additional contextual data, such as meta-information about camera settings or shooting conditions.

This paper discusses current NN-based approaches for noise estimation, suppression, classification, camera characterization, and image source identification. Special attention is paid to CNN-based extraction of camera noise footprints. Advantages and limitations of NN-based methods over traditional algorithms are analyzed.

The paper is structured as follows. In Section 2, camera noise is defined and traditional methods of its estimation are presented. Section 3 describes the main features of NN-based approaches. In Section 4, the application of NN-based methods for noise estimation, suppression, camera identification, etc., are described. The results are discussed in Section 5 and are further evaluated in Section 6.

## 2. Digital Camera Noise

### 2.1. Types of Camera Photosensor Noise

Digital camera noise refers to the distortions introduced into the image during the registration process. These distortions occur due to the imperfections in the camera photosensor, its internal processes, and the physical basis of the registration process itself. It is necessary to distinguish the concepts of the camera noise and noise of the registered image. Image noise is defined as distortions of the already registered image. In this case, digital camera noise is one of the components of image noise. Additionally, image noise depends on such factors as changes in illumination during exposure, changes in camera position, variations in quantum efficiency for different light wavelengths, and others. These factors and the imperfections of current noise estimation algorithms (e.g., from a single image [7]) can lead to other noise parameters being more impactful than camera noise.

Nowadays, various standards are used to measure and characterize digital camera noise. One widely used standard was proposed by the European Machine Vision Association (EMVA) known as the EMVA 1288 standard [8]. This standard describes all noise characteristics of digital photosensors using four parameters. Two of the parameters describe dark noise. They characterize the noise present without external illumination. The other two describe light noise. They depend on the parameters of external illumination of the photosensor. Each type is further separated according to the axis along which the signal is fluctuating. Noise arising from signal fluctuations in time is called temporal, and noise arising from differences in signal from pixel to pixel is called spatial. Thus, camera noise is categorized into the following four components:

(1) Dark temporal noise.
(2) Dark spatial noise.
(3) Light temporal noise.
(4) Light spatial noise.

**Figure 1:**
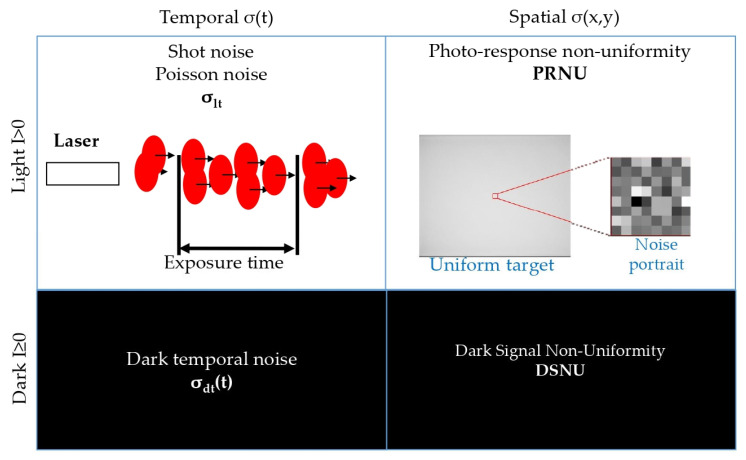
Diagram of noise characteristics according to the EMVA 1288 standard.

The main characteristics of each type of noise are shown in Figure 1. All noise components are expressed in digital number (DN)—the value of the digital signal at the camera output or in the number of generated charges (electrons). To simplify the equation representations, we will note the noise components in DN.

Dark temporal noise σ_dt_ is the temporal signal change in the absence of light on the photosensor. Dark temporal noise is caused by dark currents, random charge generation processes in the pixels, etc. Dark spatial noise σ_ds_ is the Dark Signal Non-Uniformity (DSNU). DSNU characterizes the sensitivity of the photosensor to dark currents and differences in the probability of dark charge occurrence. Light temporal noise σ_lt_ is the shot photon noise. It represents the difference in the number of photons hitting the pixels, even when the average illumination is unchanged. Light temporal noise has a Poisson noise statistic representation [9]. Another factor connecting the light temporal noise and the signal *S* is the overall system gain of conversion gain constant *K* (DN to electrons conversion coefficient) that depends on the bit depth of the camera. The light temporal noise can be written in DN as follows:

(1) $$\sigma_{lt\_DN}=\sqrt{S_{DN}\cdot K_{DN/e}}$$

where σ*_lt_*__*DN*_ is light temporal noise (in DNs), *S_DN_* is the signal value (in DNs), and *K_DN_*_/*e*_ is single value of the constant system gain (in units of DN to electrons ratio). The total temporal noise is as follows:

(2) $$\sigma_{t\_DN}=\sqrt{\sigma_{dt\_DN}^{2}+\sigma_{lt\_DN}^{2}}=\sqrt{\sigma_{dt\_DN}^{2}+S_{DN}\cdot K_{DN/e}}$$

where σ*_t_*__*DN*_ is total temporal noise (in DNs), and σ*_dt_*__*DN*_ is dark temporal noise (in DNs).

Light spatial noise σ_ls_ is characterized by the PRNU value. It is mainly caused by the variations in the photosensitive area of the different pixels. PRNU is usually described as fractions of the signal in a pixel. The light temporal noise in DN can be written as follows:

(3) $$\sigma_{ls\_DN}={PRNU}_{rel.un.}\cdot S_{DN}$$

where σ*_ls_*__*DN*_ is the light spatial noise (in DNs), and *PRNU_rel_un_* is the photo response non-uniformity value (in dimensionless relative units of the signal). The total spatial noise is as follows:

(4) $$\sigma_{s\_DN}=\sqrt{\sigma_{ds\_DN}^{2}+\sigma_{ls\_DN}^{2}}=\sqrt{{\left({DSNU}_{DN}\right)}^{2}+{\left({PRNU}_{rel.un.}\cdot S_{DN}\right)}^{2}}$$

where σ*_s_*__*DN*_ is total spatial noise (in DNs), σ*_dt_*__*DN*_ is dark temporal noise (in DNs), and DSNU*_DN_* is dark signal non-uniformity (in DNs).

DSNU and PRNU represent the average of spatial inhomogeneities across the entire area of the photosensor. At the same time, each pixel has its own PRNU and DSNU values, which give us the matrices for these two types of spatial noise. These matrices are unique for each photosensor and are sometimes referred to as photosensor “fingerprints”. They can be used for camera identification, i.e., for determining which camera the image was taken on or whether different images were taken by the same camera [10]. The system gain *K* can also be represented as a matrix *K*(*i*,*j*) [11]. The EMVA 1288 standard recommends that *K* be represented as a single value for the entire photosensor. In order to eliminate contradictions, we will follow the Standard recommendations. In our equations *K*, PRNU and DSNU are the average values for the whole photosensor, i.e., they are single-value (not matrix).

In addition to the four types of noise mentioned above, the quantization noise, characteristic to the digital systems, is sometimes considered separately [12]. It is digital systems specific and equal to the following (in DN):

(5) $$\sigma_{q\_DN}=0.288\;DN.$$

Therefore, the total noise of a camera photosensor can be written as follows:

(6) $$\sigma_{full}=\sqrt{\sigma_{t}^{2}+\sigma_{s}^{2}\;}=\sqrt{\sigma_{lt}^{2}+\sigma_{dt}^{2}+\sigma_{ls}^{2}+\sigma_{ds}^{2}\;}$$

In DNs, the total noise (in DN units) can be written as follows:

(7) $$\sigma_{full\_DN}=\sqrt{\sigma_{dt\_DN}^{2}+S_{DN}\cdot K_{DN/e}+{\left({DSNU}_{DN}\right)}^{2}+{\left({PRNU}_{rel.un.}\cdot S_{DN}\right)}^{2}}$$

**Figure 2:**
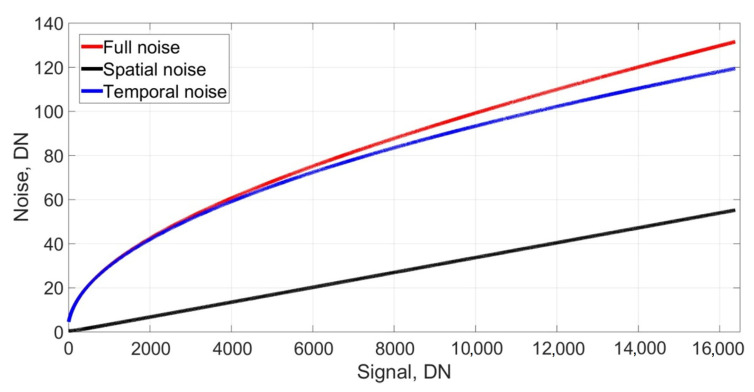
Dependencies of spatial, temporal, and total noise vs. the signal level for the camera Retiga R6.

Examples of the noise parameters for three cameras are given in Table 1. The cameras have different application areas and are machine vision PixeLink PL-B781F (PixeLink, Ottawa, ON, Canada), microscope Retiga R6 (QImaging, Surrey, BC, Canada), and mirrorless Canon EOS M100 (Canon, Oita, Japan) cameras [13]. Dependences of total, temporal, and spatial noise vs. signal for the camera Retiga R6 are shown in Figure 2.

Simplified noise models are also commonly used. They are named after their respective distributions: Gaussian [14], Gaussian–Gaussian [15], Poisson–Gaussian [16] and Poisson model [17]. The Gaussian–Gaussian model uses the sum of two Gaussian functions to describe the distribution of noise across the photosensor. The Poisson–Gaussian model uses the sum of the Poisson and Gaussian distributions. These models are actively used for noise modeling in generated images [18,19], single image noise estimation [19,20,21,22], etc. Some analogy can be found between the EMVA 1288 standard and Gaussian–Gaussian and Poisson–Gaussian models. The light temporal noise in the standard and Poisson–Gaussian model is described by a Poisson distribution. In the Gaussian–Gaussian model, it is one of the Gaussian distributions, which can be used instead of a Poisson distribution at high signal levels. In the Poisson–Gaussian model, the remaining noise (dark and light spatial noise) is described by a Gaussian distribution, and in the Gaussian–Gaussian model it is a second Gaussian. In the Gaussian and Poisson models, all noise is considered as one Gaussian or Poisson distribution. It can be seen that in the Gaussian, Gaussian–Gaussian, Poisson–Gaussian and Poisson models, there is no separation into four types of noise. However, in many cases it is important to have information about the individual noise parameters.

### 2.2. Interconnection of Applications

**Figure 3:**
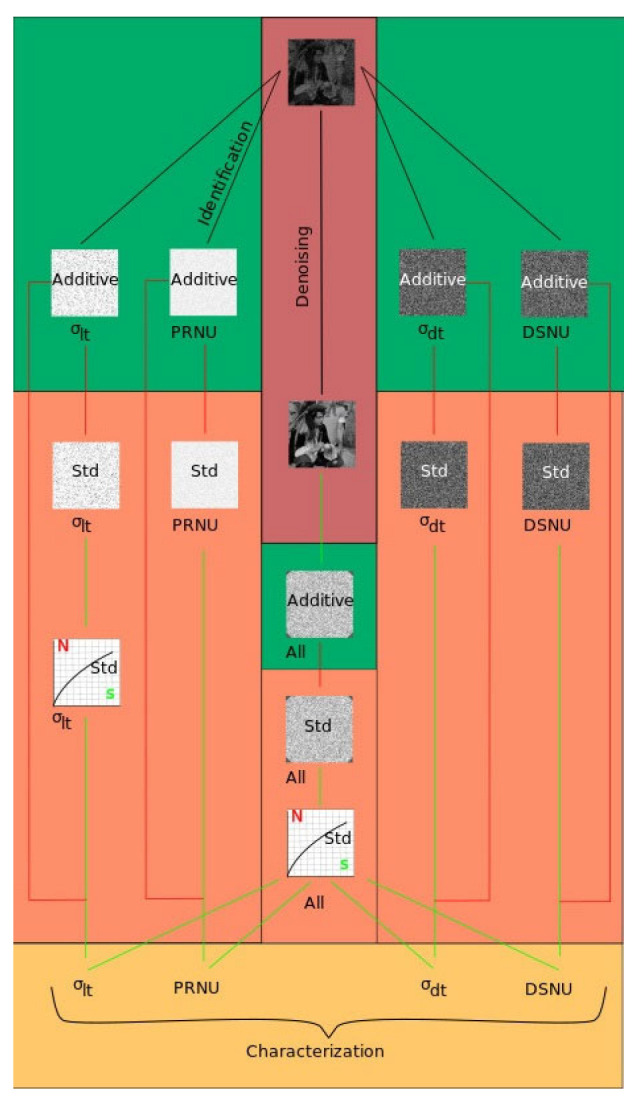
Diagram of digital camera noise data representation.

Digital camera noise information can be used for various applications: device selection, camera identification, characterization, noise reduction, etc. To demonstrate the noise representation, a diagram is presented in Figure 3. The green boxes show additive noise matrices (matrices of noises). Values in each pixel are added to the ideal signal. Additive noise values in the matrices can be positive, negative or zero. The orange boxes show noise standard deviation (Std) values (matrices of noise Std). Values of the noise Std in the matrices are non-negative. Also, dependency of the light temporal noise vs. the signal that is the 1D matrix with Std values is located here. The yellow box shows single averaged PRNU, DSNU, dark temporal, and light temporal noise values. The red box indicates the registered image that can be used for various tasks and noiseless image after ideal denoising. Green arrows indicate transitions between tasks that can be performed both in direct and inverse directions. Red arrows indicate one-way transitions and transformations.

It can be seen that the most preferred option is to obtain all four noise matrices: PRNU(*i*,*j*), light temporal σ_lt_(*i*,*j*), dark temporal σ_dt_(*i*,*j*), and DSNU(*i*,*j*). This is the most complete and informative form of noise estimation. It can be successfully used for all applications: photosensor characterization, camera selection, source identification (based on the estimation of the PRNU(*i*,*j*) matrix), and noise suppression (based on the compensation of the noise matrices). However, the problem of obtaining all four matrices from a single image is extremely difficult and currently unsolved. So, simplified options are usually considered. For example, for camera identification, the most important part is the PRNU(*i*,*j*) matrix estimation. For noise suppression, the noiseless image can be used; however, obtaining the noiseless image is also a difficult task which has not yet been solved, either. Various filtration methods require some image noise information. In some methods, pre-filtering is used, estimating noise by finding the standard deviations of the full noise at all signal levels. This allows us to estimate the camera noise parameters, albeit with low accuracy. After pre-filtering, the noise information is used for the filtering method.

**Figure 4:**
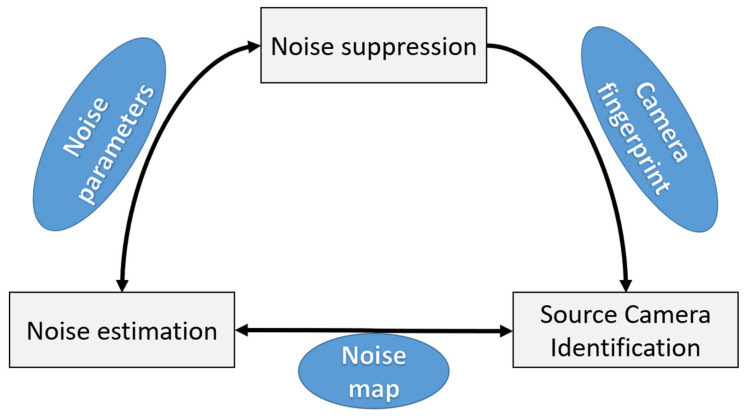
Interconnectivity between different practical applications and camera and noise parameters.

Another option for noise estimation from an image is the direct measurement of the four values characterizing noise: PRNU, DSNU, *K*, and dark temporal noise [13]. This can be used to obtain the correlation between signal and noise and standard deviation matrices in a given image. This approach is suitable for both camera characterization and noise suppression, and other tasks (see Figure 4).

### 2.3. Noise Estimation for Characterization, Denoising, and Identification

The EMVA 1288 [8] standard is a robust algorithm for accurate estimation of all noise parameters as well as for radiometric characterization. However, it is challenging to implement in real systems [23]. A special uniform scene is required that fulfills the following criteria:

(8) $$\frac{E_{max}-E_{min}}{\mu_{E}}\cdot 100<3\%$$

where *E_max_*, *μ_E_*, and *E_min_* are the maximum, average, and minimum signal in the scene. It is recommended to use a light source with the full width at half maximum, less than 10 nm. Therefore, it is required to assemble a special setup to provide the necessary uniformity of the recorded scene. It is recommended to register at least 50 sets of images (at least two images each). The sets should be registered such that the average frame signal level is distributed evenly from the minimum to the maximum camera signal. Processing of the captured images starts with the calculation of dark temporal and dark spatial noise:

(9) $$\sigma_{DTN}=\sqrt{\frac{\sum_{i,j}{\left(D_{1}\left(i,j\right)-D_{2}\left(i,j\right)\right)}^{2}}{2MN}}$$

(10) $$DSNU=\sqrt{\frac{\sum_{i,j}{\left(\bar{D(i,j)}-\bar{D}\right)}^{2}}{\left(MN-1\right)}-\sigma_{DTN}^{2}}$$

(11) $$\bar{D(i,j)}=\frac{D_{1}\left(i,j\right)+D_{2}\left(i,j\right)}{2}$$

(12) $$\bar{D}=\frac{\sum_{i,j}\bar{D(i,j)}}{MN}$$

where *D*_1_,_2_(*i*,*j*)—dark images taken without external illumination, *M*, *N*—their dimensions, *i*,*j*—row and column coordinates.

Next, the light temporal noise and the PRNU value can be calculated:

(13) $$\sigma_{LTN}=\sqrt{\frac{\sum_{i,j}{\left(L_{1}\left(i,j\right)-L_{2}\left(i,j\right)\right)}^{2}}{2MN}-\sigma_{DTN}^{2}}$$

(14) $$PRNU=\frac{\sqrt{\frac{\sum_{i,j}{\left(\bar{L(i,j)}-\bar{L}\right)}^{2}}{\left(MN-1\right)}-\sigma_{LTN}^{2}-\sigma_{DTN}^{2}-{DSNU}^{2}}}{\bar{L}-\bar{D}}$$

(15) $$\bar{L(i,j)}=\frac{L_{1}\left(i,j\right)+L_{2}\left(i,j\right)}{2}$$

(16) $$\bar{L}=\frac{\sum_{i,j}\bar{L(i,j)}}{MN}$$

where *L*_1,2_(*i*,*j*) are light images taken with external illumination.

**Figure 5:**
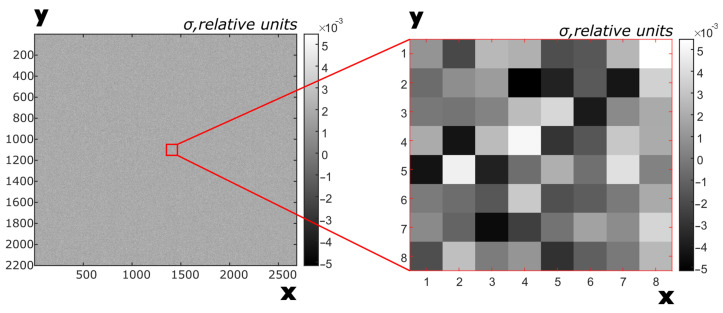
Example of a photo response non-uniformity of photosensor (PRNU matrix).

Within the framework of the EMVA 1288 standard, DSNU(*i*,*j*) and PRNU(*i*,*j*) matrices can be measured. This requires at least 100 dark images and at least 100 light images to be registered at the average signal level of the photosensor. This number of shots is necessary to reduce the impact of the temporal noise. After that, the DSNU(*i*,*j*) and PRNU(*i*,*j*) matrices can be obtained:

(17) $$DSNU\left(i,j\right)=\bar{D_{N}\left(i,j\right)}-\bar{D},\;\bar{D}=\frac{\sum_{i,j}\bar{D_{N}(i,j)}}{MN},\;\bar{D_{N}(i,j)}=\frac{\sum_{k=1}^{N}D_{k}}{N},$$

(18) $$PRNU\left(i,j\right)=\frac{\bar{L_{N}\left(i,j\right)}-\bar{D_{N}\left(i,j\right)}}{\bar{L}},\;\bar{L}=\frac{\sum_{i,j}\bar{L_{N}(i,j)}}{MN},\;\bar{L_{N}(i,j)}=\frac{\sum_{k=1}^{N}L_{k}}{N}$$

where *N* is the number of images, and *k* is the image index. An example of a PRNU(*i*,*j*) matrix is shown in Figure 5.

Since the EMVA 1288 standard is very labor-intensive, other approaches that require a smaller number of images have been developed. Methods for fast and accurate noise estimation are based on automatic scene segmentation [13]. Segmentation involves sorting signals and their corresponding deviations between images. Automatic segmentation has been successfully applied to inhomogeneous [24], homogeneous [25], and bandpass [26] scenes. Segmentation of an inhomogeneous [24] scene allows for estimation of temporal noise from two shots, but it cannot correctly estimate spatial noise. The homogeneous scene segmentation [25] gives an accurate estimate of all four noise components over four shots: two homogeneous and two dark frames. Strip scene segmentation [26] estimates all four noise components from two shots with four stripes. The noise estimates obtained by these methods can be used for every application: characterization, camera comparison, identification, noise reduction, etc.

A variety of methods for noise suppression have been developed. They estimate the noise from a single image, which then will be filtered. These methods can be separated into three main groups: filtration-, wavelet transformation-, and patch-based. Filtration-based methods are the least complex [27,28,29,30]. By subtracting the filtered image from the image and removing the edges in the resulting image, noise can be estimated. Considering the challenges of parameter selection for both steps of the algorithm, obtaining a reliable filtration-based noise estimation method is an extremely difficult task.

A more sophisticated approach is based on wavelet transformation. The resulting wavelet coefficients are used for noise estimation [31,32,33,34,35]. However, the residual image edges degrade the noise estimation quality. To reduce their impact, in [33] edges were removed, and in [35] an upper signal threshold was implemented.

Currently the most promising approach is based on image patches [36,37,38,39,40,41,42,43]. Each image is divided into patches. Signal fluctuations in each patch are assumed to only be caused by noise. In [36,37], threshold selection is applied to select the most uniform patches. In [39], the eigenvalues of the covariance matrix in redundant dimensions were calculated to estimate the noise.

For camera identification [44], the two main goals are to determine whether images were taken by a single camera and whether an image was taken by a specific camera. Using smoothing filtration, the high frequency component of the image is identified. Dividing it by the original image allows us to obtain the relative deviation magnitudes (image portrait). To identify whether images were taken using a single camera, the noise estimates from different images are compared (e.g., using the correlation coefficient). Although noise estimation is not particularly accurate, it is usually enough to reliably determine whether the images were taken with the same camera.

To determine whether an image was taken by a specific camera, it is necessary to measure its spatial noise characteristics. For this, hundreds of homogeneous light and dark images are recorded. The light images are averaged (to suppress the temporal noise), and the averaged dark image is then subtracted and divided by the average signal. This allows us to obtain the PRNU(*i*,*j*) matrix. The image portrait is then numerically compared to this noise portrait. This approach has been shown to be effective even in the case of JPG compression, which indicates its reliability and efficiency.

## 3. Fundamentals of Neural Network-Based Image Processing

### 3.1. Training of Neural Networks

NNs require training before they can be used. Training is a process of nonlinear optimization of the NN’s parameters. The difference between the NN’s output and the expected result is calculated using a loss function [45]. For noise estimation and processing, both supervised and unsupervised training can be applied [1].

The training datasets can be separated into real and synthetic datasets. Quality of training datasets for NN processing of noise depends on the variety of models used to generate data, noise levels, dataset size, and the resolution of individual images. Real datasets contain images captured with pre-calibrated photosensors. The calibration process and the reference noise extraction are typically very resource- and time-intensive. This can limit the size of commonly used datasets such as DID [46], SIDD [47], DND [48], SID [49], ELD [50], BSD300 [51], McMaster [52], BSD500 [53], Nam [54], and others.

The lack of available real training data can partially be addressed by numerical noise models, including NN-based [55,56,57,58,59,60,61,62,63,64,65,66] noise modeling techniques. Synthetic datasets are usually generated by adding noise to noise-free images, which allows us to precisely control all aspects of the noise in each image. This helps create optimal numerical models for rapid data generation that can be used for NN training. However, the properties and limitations of numerical noise models can also decrease the training efficiency.

### 3.2. Convolutional Neural Networks

**Figure 6:**
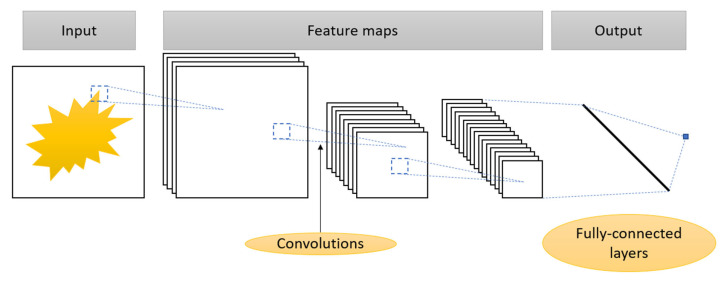
Example of a convolutional neural network architecture.

Convolutional neural networks are the most common type of NN for image processing and noise suppression applications [1]. Convolutional neural networks are a class of deep neural networks inspired by the structure of the visual cortex of the brain [62]. CNNs outperform fully connected NNs in processing data with topological structure, such as images, video, audio, and other types of multidimensional signals. A typical structure of convolutional neural networks is shown in Figure 6.

In contrast to fully connected NNs, where each neuron in one layer is connected to all neurons in the previous layer, in CNNs each neuron is connected only to a small local area in the previous layer—the so-called local receptive field [63]. This structure reflects the biological organization of the visual cortex, where neurons respond to stimuli in a limited part of the visual field [62]. This reduces the number of training parameters and makes the NN output dependent on the spatially localized features such as edges, shapes or textures. Another feature of CNNs is the use of identical parameters throughout the input layer to compute the activation function in a single receptive field. These common weights form the convolution kernel, which serves as a filter for extracting certain types of local features regardless of their position in the image. This ensures translational invariance, which allows us to form a map of feature distributions across the image [63,64].

The basic structure of a CNN architecture includes a sequence of alternating convolutional and pooling layers. The input layer accepts multidimensional data, such as a “width × height × number of channels” tensors for RGB images. The output of the convolution layer can be expressed as follows:

(19) $$y(i,j)=\sum_{m}\sum_{n}x(i+m,j+n)\cdot k(m,n)$$

where x(i, j) is the input image, k(m, n) is the convolution kernel, and i, j and m, n are the indices of the output matrix and the convolution kernel, respectively. Each kernel forms its own feature map and several kernels form the output tensor with the corresponding number of channels. The size of the output feature map is determined by the input parameters, the kernel size, the convolution step, and the edge processing (padding) [63,65].

Rectified Linear Unit (ReLU, f(x) = max(0, x)) is the most commonly used activation function in CNNs [66]. ReLU has low computational complexity, is effective against vanishing gradients, and can be biologically interpreted.

The current development of CNNs, such as VGG [65], ResNet [67], and Inception [68], is built on improving the depth and organization of layers, introducing attention mechanisms, and optimizing information flow while maintaining the basic principles.

**Figure 7:**
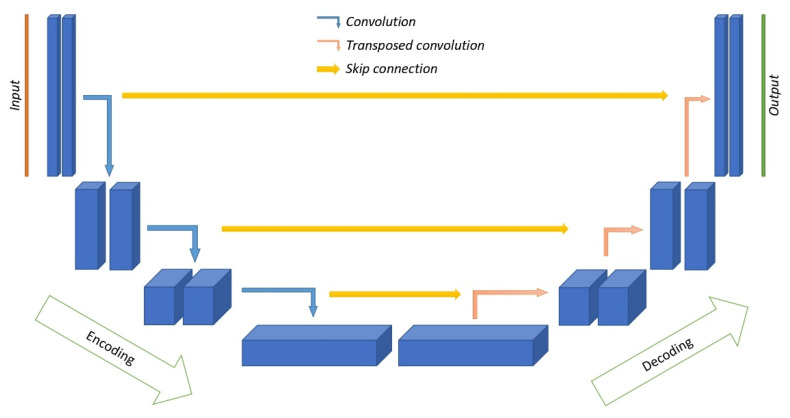
The U-Net architecture.

Currently, the most commonly used architecture for image processing [69] is the U-net architecture, developed in 2015 for segmentation of biomedical images [70]. The main elements are shown in Figure 7.

The U-Net is named after its characteristic U-shape and consists of two symmetric parts: the encoder and the decoder. The encoder is responsible for feature extraction and compression of spatial information. The encoder forms the hierarchy of features. The initial layers extract simple features such as edges, corners, and homogeneous regions. These feature maps have high spatial resolution but contain relatively few channels. In the middle layers, simple features are combined into more complex features such as geometric shapes, parts of objects, and characteristic textures. Here the resolution decreases and the number of channels increase. In the last layers, high-level, semantic representations are formed—complete objects and their characteristic combinations. The resulting feature maps have minimal spatial resolution but a large number of channels, reflecting the abstract properties of the input data [63,65]. The feature extraction is performed by the encoder blocks which include two consecutive convolution operations each and a ReLU activation function.

The convolution operation is followed by a pooling operation, which performs spatial subsampling, i.e., reducing the size of the feature map. The most commonly used pooling layers are max pooling, which selects the most pronounced features, and average pooling, which provides smoothing [64]. Pooling reduces computational complexity, reduces the risk of overtraining, and increases the invariance of the network to scale and affine transformations of the input data. Pooling has no trainable parameters [64]. The purpose of the compression branch is to reduce the spatial resolution of feature maps and increase the number of channels to extract abstract high-level features.

The decoder performs the opposite operation—the resolution of feature maps is increased in order to restore accurate localization of objects. Here, at each decoder block the size of the feature maps increases, while the number of channels is reduced. The output of each decoder block is also concatenated with the corresponding feature maps from the encoder. These skip-connections allow the integration of high-level semantic information with high spatial resolution recovered from the initial layers of the encoder. The skip-connections not only compensate for the loss of spatial information due to pooling but also provide integration of contextual and localized information. As a result, pixel classification relies not only on the properties of the full scene but also on localized features.

### 3.3. Generative-Adversarial Network Architecture

**Figure 8:**
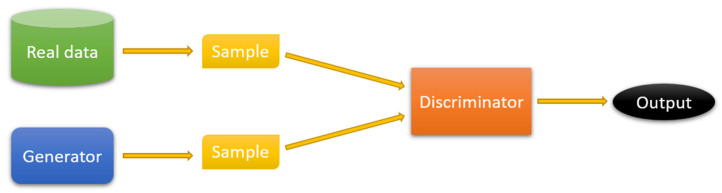
Example of a GAN model.

Generative-adversarial networks (GANs) are a class of neural network algorithms based on the training of two CNNs at the same time. The GAN model implements an adversarial model between two NNs, a generator and a discriminator. The purpose of the generator is to create synthetic samples that mimic real data. In turn, the discriminator has to distinguish between the real samples and samples created by the generator. An example of a GAN model is shown in Figure 8.

The training of a GAN model is interpreted as an optimization problem with a minimax loss function, where the discriminator seeks to maximize the probability of correct classification and the generator seeks to minimize the probability of the discriminator detecting synthetic data. This process involves updating the parameters of each network in turn. First, the generator’s parameters are assumed to be unchanged, and the discriminator is trained to distinguish between real and synthetic data based on batches containing both types of samples. The generator then optimizes its parameters to overcome the current discriminator parameters. It is critical to maintain balance between the generator and the discriminator. If one part significantly outperforms the other, the overall performance of the model can degrade.

In the context of NN noise processing, there are GAN models for synthesizing noisy images with spatial noise correlation [71,72], as well as models for noise suppression [73].

## 4. Practical Applications of Neural Network Methods

### 4.1. Synthesis of Datasets and Noise Modeling

**Figure 9:**
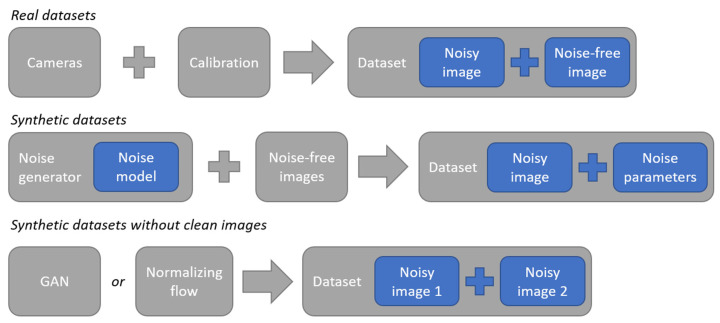
Different types of noise datasets that can be used for neural network training.

Synthetic datasets for NN training can save time and resources on preparing real datasets of noisy and noise-free images [74] (see Figure 9). At the same time, synthetic datasets are often used for noise estimation [55]. This is demonstrated by the compact NoiseFlow model, which has fewer than 2500 parameters. It is one of the first NN-based models for camera sensor noise estimation [56]. NoiseFlow uses conditional normalizing flow to model complex signal-dependent and gain-dependent noise beyond parametric representations. The model was trained and evaluated on the SIDD dataset [47], which contains approximately 30,000 thousand raw–RGB image pairs from five smartphone cameras, captured under various ISO exposure levels (50–10,000) and lighting conditions (low, normal, and high brightness; 3200 K, 4400 K, and 5500 K color temperature). The dataset covers a range of noise levels, with the standard deviation of Gaussian noise of approximately 1.62 to 23.5. On this dataset, NoiseFlow achieved a negative log-likelihood (NLL) of −3.521 nats/pixel on the test set. This represents a 0.42 nats/pixel improvement (or a 51.6% increase in likelihood) over the camera-calibrated noise level functions (NLF) and a 0.69 nats/pixel improvement (a 99.4% increase in likelihood) over a homoscedastic additive white Gaussian noise (AWGN) model. Furthermore, noise samples generated by the NoiseFlow exhibited a significantly lower marginal Kullback–Leibler (KL) divergence (0.008) from the real noise compared to the camera NLF (0.052) and AWGN (0.394) baselines. In similar noise levels (e.g., medium to high noise conditions with standard deviation values between 4.79 and 23.5), the model’s superior performance in both density estimation and noise synthesis demonstrates its ability to capture the complex, non-Gaussian characteristics of real photosensor noise more accurately than traditional parametric models. However, the model’s disadvantages include its primary design for raw–RGB sensor data, making it less suitable for processed sRGB images where the noise distribution is altered by the camera’s image signal processor. Additionally, the computational complexity of inverting normalizing flows for sampling could pose challenges in memory- or latency-constrained scenarios.

The concept of NN-based noise generators has evolved with the development of the CANGAN framework, which takes into account camera features by training on real photosensor noise [57]. The CANGAN framework consists of two parts: the U-Net [70]-based Noise-Generator Network and the Camera-Encoder Network. The Camera-Encoder Network is used to extract a camera-specific latent vector from a noisy image, while the Noise-Generator Network can mimic the camera noise based on a clean image and camera parameters. This allows the model to generate noise for a particular camera sensor. The proposed method was trained and evaluated on approximately 24,000 raw–RGB image pairs from the SIDD dataset [47], captured under various ISO exposure levels (from 50 to 10,000) and lighting conditions (low and normal illumination). Quantitative evaluation demonstrated that CANGAN significantly outperforms existing models, achieving a KL divergence of 0.00159, which represents a 74.7% improvement over the Poisson–Gaussian model (0.01006) and a 72.0% improvement over the NoiseFlow model (0.00912) [56]. Similarly to NoiseFlow [56], CANGAN’s lower KL divergence indicates a superior ability to capture the complex, camera-specific noise distributions present in real data. When the CANGAN was used to generate training data for the DnCNN [75] denoiser, it achieved peak signal-to-noise ratio (PSNR) [76] of 48.71 dB and structural similarity index measure (SSIM) [77,78] of 0.992, outperforming the same denoiser trained on NoiseFlow-generated data (48.52 dB, 0.991) and real data (48.30 dB, 0.989). On the other hand, an important disadvantage of this approach is its reliance on a camera encoder that requires a sample noisy image from the target camera in order to extract the latent vector, which may limit its applicability in scenarios when a sample cannot be obtained. Furthermore, the adversarial training framework and the U-Net [70] architecture of the generator introduce significant computational complexity and memory demands compared to simpler parametric models.

Subsequently, a contrast training framework was proposed to estimate fine-grain camera noise parameters, such as the variance of light shot noise or readout noise, from a single raw image [58]. This ResNet-based [67] framework employs a contrastive learning strategy to train a noise estimation model. It is trained on a dataset of carefully calibrated camera sensors (including Canon EOS 5D4, Nikon D850, Sony RX100VI, and HUAWEI P40 Pro), where noise parameters for each image are represented as a four-tuple $\left(K,\;\sigma ,\;\mu_{c},\;\sigma_{r}\right)$, encompassing overall system gain K, readout noise standard deviation σ, mean readout noise (or color bias) $\mu_{c}$, and row noise standard deviation $\sigma_{r}$. The model uses contrastive loss to maximize agreement between augmented views derived from the same noise parameters. This enables it to separate and accurately estimate the components of a complex, physics-based noise model that includes signal-dependent Poisson shot noise and multiple signal-independent Gaussian noise sources. The estimated noise parameters are used to define a joint distribution as a base for sampling and generation of realistic training data. This generated data, when used to train a denoiser, achieved a competitive PSNR of 50.13 dB and SSIM of 0.9891 on the SIDD benchmark, outperforming methods based on AWGN (47.55 dB, 0.9698), Poisson–Gaussian (49.91 dB, 0.9896), NoiseFlow (44.96 dB, 0.9517) [56], and CANGAN (49.19 dB, 0.9879) [57], and approaching the performance of training with real paired data (50.10 dB, 0.9902). The proposed fine-grained model’s superior parameter estimation leads to synthetic noise that closely matches the real noise distribution. This is quantitatively reflected in the lower KL divergence achieved by the synthesis pipeline (average of 0.0211 across five SIDD cameras) compared to AWGN (0.7544), Poisson–Gaussian (0.0467), NoiseFlow (0.0590) [56], and CANGAN (0.0220) [57]. However, the current restriction to Bayer color filter arrays (CFAs) limits the approach, making it unsuitable for sensors with alternative CFAs like RYYB, RGBC, RGBW, RWB or X-Trans. Furthermore, while the original paper does not provide explicit computational metrics, the complexity of the contrastive learning framework and the deep ResNet [67] backbone suggests the associated computational costs for training are expected to be high.

In [59], an unsupervised GAN training framework C2N for noise modeling was proposed. The C2N does not require the preparation of the training dataset of noisy and noise-free images. Instead, it trains using unpaired sets of clean images and real noisy images. This approach explicitly separates the noise generation process into dedicated modules for signal-dependent and signal-independent components, further enhanced by convolutional transformations to model spatial correlations inherent in real camera noise. The framework was trained and evaluated on real-world benchmarks including SIDD [47] and DND [48] datasets. Quantitative results demonstrate that a denoiser trained with data generated by the C2N significantly outperforms other unsupervised methods, achieving a PSNR of 33.76 dB and SSIM of 0.901 on the SIDD benchmark with a DnCNN [75] backbone, and 35.35 dB/0.937 with a larger DIDN [79] backbone. This represents an improvement of over 1 dB in PSNR compared to previous generation methods like UIDNet (32.48 dB) [80]. In the complex real-world noise conditions present in the SIDD [47] and DND [48] benchmarks, which include signal-dependent (e.g., photon shot noise) and signal-independent components with spatial correlations, the C2N’s modular design enables it to more accurately capture the true noise distribution. This is quantitatively supported by its lower KL divergence (0.1638) compared to AWGN (0.1746) when comparing generated noise to the ground truth. A key disadvantage of this approach is the framework’s inherent instability during adversarial training, which can be partially mitigated by the proposed stabilizing loss term to prevent color shifting. In addition, while the C2N eliminates the need for paired data, its performance is still contingent on having access to a representative set of unpaired clean and noisy images from the target domain. Finally, the computational complexity of the framework’s architecture is not explicitly specified and heavily depends on the backbone denoiser used.

The emergence of unsupervised NN models has led to the creation of Noise2NoiseFlow [60] based on the Noise2Noise model [61] with integrated normalizing flow. The Noise2NoiseFlow training uses pairs consisting of two independent noisy images of the same scene, instead of the traditional pairs of noisy and noise-free images, thereby eliminating the need for clean ground truth data. The framework was evaluated on the SIDD dataset [47], utilizing approximately 500,000 patches 32 × 32 pixels each, with ISO exposure levels from 100 to 3200 with a 70/30 train/test split. The jointly trained model achieved NLL of −3.501 nats/dim and a KL divergence of 0.0265 on the test set. This makes the Noise2NoiseFlow performance on par with the fully supervised NoiseFlow model (NLL: −3.502, KL: 0.0267) and significantly superior to statistical baselines like the camera NLF (NLL: −3.282, KL: 0.0578) and AWGN (NLL: −2.874, KL: 0.4815). Furthermore, the integrated DnCNN [75] denoiser achieved a PSNR of 52.80 dB and SSIM of 0.984, outperforming a DnCNN trained with standard Noise2Noise (51.57 dB) and even a supervised DnCNN trained on real clean/noisy pairs (51.32 dB). Despite its advantages, the method requires precisely aligned pairs of noisy images from the same scene. This requirement poses an additional challenge for practical applications.

The DCD-Net [81] was proposed in order to address the lack of quality training data. DCD-Net is a system with an iterative “denoise—corrupt (add synthetic noise)—denoise” algorithm. For each input image, the system iteratively suppresses the noise, estimates the noise characteristics, and adds the noise back. Repeating this cycle, the system can train only on noisy images, without any noise-free samples. The proposed method was evaluated on both synthetic and real-world benchmarks. On synthetic sRGB datasets (Kodak, BSD300 [51], Set14 [82]) with Gaussian noise, DCD-Net achieved a PSNR/SSIM of 32.27/0.881, 31.01/0.876, and 31.29/0.862, respectively, closely matching the performance of the strongly supervised Noise2Noise [61] baseline. For real-world denoising, on the SIDD [47] raw–RGB validation set, it achieved a PSNR of 51.40 dB and a SSIM of 0.992, surpassing other self-supervised methods, including Blind2Unblind [83] which requires 17 times more computational resources. The method assumes the noise can be modeled by a Poisson–Gaussian distribution and employs a dedicated noise estimation network trained with a patch variance loss to predict a pixel-wise noise level map. However, the framework’s iterative nature increases its computational cost up to three times when compared to a standard U-Net architecture [70]. Furthermore, its performance is contingent on the accuracy of the initial blind-spot network used for bootstrapping and may degrade if the underlying noise deviates significantly from the assumed heteroscedastic Gaussian model.

In [74], further improvements to contrast training approach were made, resulting in a noise estimation and generation framework that does not require pre-calibrated sensors. Similarly to [58], the framework is based on a fine-grained physics-based noise model that includes shot noise, readout noise, row noise, and color bias. The proposed framework was trained using a specialized data augmentation strategy that generates diverse positive and negative samples by selectively varying individual noise parameters while ensuring distinct scene content. It achieved state-of-the-art noise parameter estimation performance. This approach enables the model to focus accurately on different noise components, enhancing its discriminative capability and robustness. The method achieves a mean absolute error of 0.1598 for color bias $\mu_{c}$ and 0.8621 for row noise variance $\sigma_{r}$ on the SIDD [49] dataset. On the SIDD dataset [47], the synthesized noise achieved an average KL divergence of 0.0198 across five smartphone cameras, outperforming statistical models (0.0285 for AWGN and 0.0247 for Poisson–Gaussian) and learning-based methods (0.0223 for NoiseFlow [56] and 0.0215 for CANGAN [57]) that require in-camera parameters or paired training data. Using this pipeline to generate training data for the denoiser, the U-Net [70] reached a PSNR of 51.40 dB on the SIDD [47] validation set, exceeding methods trained on other synthetic data such as AWGN (49.01 dB), Poisson–Gaussian (49.93 dB), NoiseFlow [56] (50.54 dB), CANGAN [57] (50.12 dB), and SINE [39] (50.77 dB), and matching models trained on real paired data (51.42 dB). The superior PSNR results confirm that training with more realistic, fine-grained noise synthesis improves denoising performance across different noise levels, particularly in challenging real-world conditions. However, the model’s complexity is substantial at 6.10 GFLOPs with 12.34M parameters. Similarly to the previous model [58], the proposed pipeline is currently limited in applicability and is not designed to accommodate CFA patterns other than the Bayer CFA.

Table 2 presents the summary of methods discussed in this section.

### 4.2. Denoising

**Figure 10:**
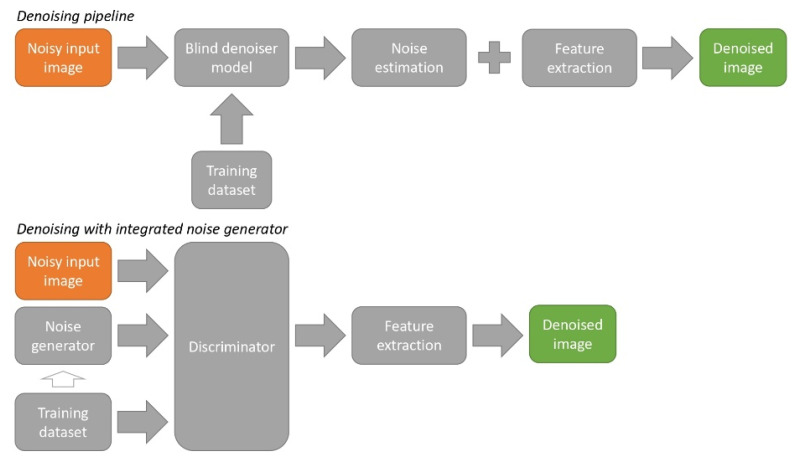
Noise suppression pipeline using neural networks.

NN-based noise suppression methods explicitly or implicitly determine noise properties during operation, often taking into account the signal level (see Figure 10). In this case, the noise estimation can be obtained both for individual pixels and for the whole image. NNs explicitly estimating the noise parameters can extract several spatial pixel features [84]. Typical examples of noise-aware CNNs with explicit noise estimation are DnCNN [75] and ADNet [85], modifications of the U-net architecture: DANet [70,86]. Accurate estimators that take into account the nature of the noise can improve the efficiency of noise suppression algorithms [87]. However, image noise is usually not separated from camera noise in noise suppression applications.

One of the first NNs to perform signal-dependent noise estimation for each pixel is DRNE [88]. Building on earlier studies, the authors assume the statistical noise distribution to be Gaussian with variance

(20) $$\sigma^{2}=\sigma_{r}^{2}+\sigma_{s}y_{p}$$

where $\sigma_{r}$ is readout noise, $\sigma_{s}$ is shot light noise, and $y_{p}$ is the true illumination intensity. This model explicitly captures the signal-dependent nature of noise, where noise intensity increases with scene brightness. The DRNE is a deep convolutional network consisting of 16 layers with 64 channels each, carefully designed without pooling or interpolation layers to preserve image details. The DRNE takes noisy RGB images as input and outputs a pixelwise noise variance map reflecting the spatially varying, signal-dependent noise levels. Quantitative evaluations were conducted on three image datasets—Kodak (24 images), McMaster [52] (18 images), BSD500 [53] (500 images)—using both homogeneous and inhomogeneous synthetic Gaussian noise.

For homogeneous noise with fixed variance, the DRNE achieved average noise estimation error competitive with the state-of-the-art SINE method [39]. For example, on the Kodak dataset at noise level σ=5, the DRNE achieved an average error of 0.08 dB compared to the SINE’s 0.09 dB. On BSD500 at σ=0, the DRNE scored 0.32 dB average error vs. the SINE’s 0.54 dB. These results demonstrate that the DRNE performs on par with leading scalar estimation methods even under the simplified homogeneous noise assumption. For inhomogeneous noise, where images were synthetically divided into four parts with different noise levels, the DRNE produced accurate pixelwise noise maps closely matching the ground truth spatial patterns. These maps revealed expected signal-dependent characteristics, such as lower noise estimates in flat image regions that contain less high-frequency content. When incorporated into deep denoising networks like the FFDNet [89], the DRNE’s pixelwise noise maps enabled significant gains in denoising performance for realistic, signal-dependent noise. For instance, on the Kodak dataset under the Poisson–Gaussian noise model, the DRNE combined with the FFDNet achieved a PSNR of 33.68 dB, surpassing the SINE method with 33.24 dB, while reducing visible artifacts in denoised images and improving visual quality. This performance gain highlights the practical benefit of using pixelwise noise estimation for denoising under more realistic, signal-dependent noise conditions. However, the DRNE’s computational demands are relatively high. Processing a 500 × 500 image took about 5.31 s on a CPU and 1.16 s on a GPU, which is considerably slower than the fastest scalar noise estimation method SINE that runs in 0.27 s on CPU. This makes the DRNE less favorable for applications requiring real-time or low-latency noise estimation. Additionally, the DRNE was trained solely on synthetic homogeneous noise with σ uniformly sampled in, which may limit its ability to generalize to more complex real-world noise that deviates from the Poisson–Gaussian assumption or exhibits spatial correlations absent from the training data. This could reduce performance on noisy images with sensor-specific or correlated noise patterns not well captured by the training distribution.

Denoising efficiency and noise estimation accuracy can be enhanced by application of frequency–domain attention mechanisms [90]. The FADNet builds upon a conventional encoder–decoder backbone by interleaving a sequence of Adaptive Instance Residual Modules (AIRMs) that fuse spatial features with multi-scale frequency cues. Additionally, input images are preprocessed via a CNN noise map extractor. The noise map extractor and frequency attention mechanism allow for better preservation of high-frequency details in the spatial spectrum. This also includes spectral noise components. The FADNet attains superior efficiency compared to contemporary denoisers. On 512 × 512 test images processed on the GPU, the FADNet requires on average 0.20 s per image to achieve 41.36 dB PSNR, whereas other methods like the CycleISP [91] need approximately 0.40 s for 40.85 dB PSNR, and the MPRNet [92] takes about 1.00 s for 40.01 dB PSNR. In terms of model size and memory footprint, FADNet comprises approximately 22 million parameters (≈88 MB model file) and peaks at ~3.0 GB of GPU memory during inference—roughly half the parameter count of the MPRNet (51 M, ≈204 MB) and 15% fewer parameters than the Restormer [93] (26 M, ≈104 MB). Computationally, the FADNet performs on the order of 150 GFLOPs per forward pass, compared to ~300 GFLOPs for the Restormer and ~500 GFLOPs for MPRNet. However, in terms of memory and computational efficiency the FADNet is still orders of magnitude more demanding than traditional non-learning-based methods which require ∼0.2 GFLOPs and less than 100 KB. The evaluation was conducted on 1200 randomly cropped 512 × 512 patches drawn from the Nam [54] and SIDD [47] benchmarks with noise levels typically quantified by standard deviation estimates ranging from approximately σ = 5 to 50 in linearized RAW space.

In addition to noisy data generation for NN training, GAN models can be used to create reference noise signals for noise suppression systems. This led to the development of a three-component noise suppression GAN model. It consists of a generator NN, a noise suppression NN based on a dual-path U-Net [70] architecture, and a discriminator NN [94]. On the SIDD dataset [47], the proposed GAN model achieved a PSNR of 39.29 dB and a SSIM of 0.915, significantly outperforming the traditional BM3D [95] method (25.67 dB PSNR, 0.687 SSIM) and learning-based TridentGAN [96] (38.40 dB PSNR, 0.904 SSIM). On the other hand, the proposed architecture exhibits significant computational demands, with the denoiser alone comprising approximately 15.6 million parameters and requiring 68.9 GFLOPs per forward pass, rendering it less suitable for deployment on resource-constrained devices. In contrast, the traditional BM3D [95] algorithm operates with minimal memory requirements of only 71 MB for 512 × 512 images and effectively zero learnable parameters, while the TridentGAN [96] achieves comparable denoising performance (38.40 dB PSNR, 0.904 SSIM) with 0.047 s inference time compared to the proposed method’s 0.051 s. Furthermore, the model’s effectiveness is contingent upon the noise characteristics; its stability may degrade with structured or non-stationary noise patterns, such as low-frequency noise, due to the fixed dilation rates and the reliance on local receptive fields.

Approaches based on direct image reconstruction as well as on denoising with a known noise level are also being developed. Examples of such NN models are RED-Net [97], RBDN [98], MemNet [99], DHDN [100], NNs based on the U-net modifications [101], and others [102,103,104]. These methods have gained attention for camera noise estimation for the purpose of image source identification.

Table 3 presents the summary of methods discussed in this section.

### 4.3. Noise Estimation for Denoising

**Figure 11:**
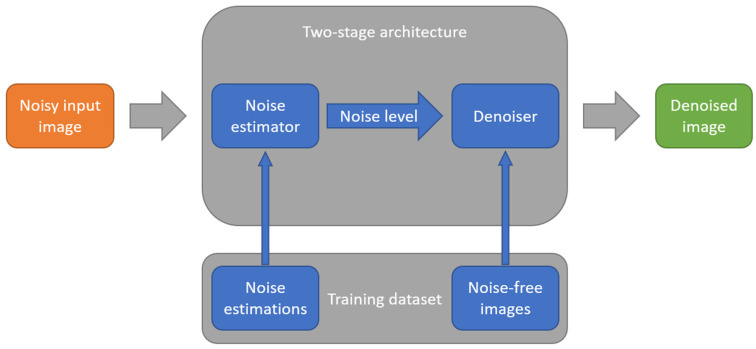
Noise suppression pipeline with two-component structure.

Camera and image noise estimation is closely related to denoising. However, NN models for these applications often require mutually exclusive modifications. Therefore, two-component systems are being developed that separate noise estimation and suppression between two NNs. An example of such a system is shown in Figure 11. These systems typically estimate image noise rather than the photosensor noise.

Noise suppression with signal-dependent noise estimation is at the core of the CBDNet model [105]. The CBDNet structure includes two subnetworks: a shallow five-layer fully convolutional network for noise estimation and a modified 16-layer U-Net [70] for non-blind denoising. The model is trained using a combined dataset of synthetic images, generated with a sophisticated noise model, and real-world noisy photographs. The synthetic noise is generated using a Poisson–Gaussian distribution that incorporates in-camera processing pipeline artifacts, including demosaicing, camera response function application, and JPEG compression. This Poisson–Gaussian distribution is approximated as a heteroscedastic Gaussian with variance

(21) $$\sigma^{2}\left(L\right)=L\cdot \sigma_{s}^{2}+\sigma_{c}^{2}$$

where L is irradiance of the pixel, $\sigma_{s}$ is signal-dependent noise variance (photon shot noise), and $\sigma_{c}$ is signal-independent noise variance (read noise). During training, the noise level parameters $\sigma_{s}$ and $\sigma_{c}$ were uniformly sampled from the ranges [0, 0.16] and [0, 0.06], respectively, to cover a wide spectrum of realistic noise intensities. For the JPEG compression scenario, the quality factor was sampled from [60, 100], representing moderate to high quality and correspondingly low to moderate compression artifacts.

The noise estimation subnetwork is trained with an asymmetric loss function and a total variation regularizer that decreases the probability of the noise underestimation during training. This approach significantly outperforms methods reliant on simplistic AWGN assumptions. On the DND dataset [48], which contains real-world sRGB images with complex, signal-dependent noise, the CBDNet achieved a PSNR of 38.06 dB and a SSIM of 0.9421, surpassing contemporary methods like the MCWNNM [106] (37.38 dB/0.9294) and the TWSC [107] (37.94 dB/0.9403). On the Nam dataset [54], which consists of JPEG-compressed images, a CBDNet variant incorporating JPEG compression achieved a PSNR of 41.31 dB and a SSIM of 0.9784. However, the model’s performance is contingent on the fidelity of its noise model to the target real-world noise; performance may degrade on images with noise characteristics not well-represented by the Poisson–Gaussian model, such as structured noise patterns.

Further development of the CBDNet architecture, which itself was influenced by the FFDNet [89], led to the NERNet model [108]. The NERNet retains the two-subnetwork structure for blind denoising but introduces significant architectural innovations to enhance performance. The noise estimation subnetwork utilizes a symmetric dilated convolution block and a pyramid feature fusion block to create a receptive field pyramid, enabling more accurate estimation of spatially variant noise levels. The noise suppression subnetwork, based on the U-Net [70], is enhanced with dense blocks and a novel Dilation Selective (DS) block incorporating an attention mechanism. This DS block adaptively fuses features from parallel convolutional layers, using a mechanism that combines local (Gram matrix-based) and global (global average pooling) attention to weigh the contributions of each branch. For synthetic noise evaluation, the model was trained and tested on standard noise levels from σ = 15 to 50. On the SIDD dataset [47], the NERNet achieved a PSNR of 37.97 dB and a SSIM of 0.942, surpassing CBDNet’s 33.28 dB and 0.868. On the Nam [54] dataset, it achieved 40.10 dB PSNR, outperforming CBDNet’s 39.01 dB. Furthermore, on synthetic noise benchmarks (BSD68 with σ=50), the NERNet reached 28.12 dB PSNR, exceeding the FFDNet [89] (27.96 dB) and the DnCNN [75] (27.92 dB).

The two-network architecture has advanced significantly with the development of the FBI-Denoiser network [109]. The FBI-Denoiser integrates two specialized neural modules: the PGE-Net, a compact convolutional regressor designed for the rapid estimation of Poisson–Gaussian noise parameters, and the FBI-Net, an efficient blind-spot network based on a modified U-Net architecture [70] that is trained exclusively on single noisy images. A critical preprocessing step involves applying the Generalized Anscombe Transformation (GAT) [110] to the input noisy image using the parameters estimated by the PGE-Net, stabilizing the noise to approximate Gaussian distribution with unit variance. This preprocessing enables the subsequent FBI-Net to perform denoising effectively in the transformed domain. The PGE-Net was trained and evaluated on images corrupted with a range of Poisson–Gaussian noise levels, with parameters (α,σ) covering very low to moderate noise intensities ((0.01, 0.0002), (0.01, 0.001), (0.05, 0.0002), and (0.05, 0.002)). The PGE-Net achieved significant speedup in noise parameter estimation compared to conventional methods, reducing the estimation time from seconds (3.123 s for Foi’s method [20] and 1.084 s for the Liu method [111] on CPU) to 0.002 s per image on the GPU when tested on BSD68 [112] (grayscale) and FiveK [113] (raw–RGB) datasets across multiple Poisson–Gaussian noise levels. This represents an approximately 1560× speedup compared to Foi’s method and a 540× speedup compared to the Liu method. The overall FBI-Denoiser pipeline—which combines fast estimation (PGE-Net) with the efficient FBI-Net blind-spot network—was evaluated on 512 × 512 real-world noisy images from the FMD [114] (microscopy), the SIDD [47] (raw–RGB), and the DND [48] (sRGB) datasets. On the DND benchmark, the FBI-Denoiser achieved PSNR of 48.02 dB and SSIM of 0.9797, demonstrating state-of-the-art performance among methods trained only on single noisy images. Under these conditions, total end-to-end inference time improves 9.5-fold, from 2.00 s per image for the BP-AIDE to 0.21 s per image for the FBI-Denoiser, while maintaining or surpassing denoising performance. Additionally, the FBI-Net uses only 340,000 parameters and 2512 MB GPU memory compared to competing blind-spot networks like the D-BSN [115] (6,612,000 parameters, 4231 MB) and the FC-AIDE [116] (754,000 parameters, 2581 MB).

The two-network models are not limited to pixel-by-pixel noise mapping. In [117], a method for global statistical noise estimation based on the PCANet [118] and the ResNet101 [119] NNs was proposed. The PCANet is used as a fragmenter defining image segments, and the ResNet101 serves as a classifier for these segments. For each segment, the σ parameter is estimated in the chi-square distribution. This method was evaluated on 100 randomly selected images from the BSD300 dataset [51], with AGWN standard deviations levels ranging from σ=5 to 40. The proposed hybrid network achieved a mean estimation error of noise standard deviation as low as 0.22, outperforming traditional methods such as [28] (0.28) and [37] (0.26). The network architecture demonstrated flat patches selecting accuracy reaching approximately 92% after training on a dataset of 1,165,600 patches.

Dual-stage denoising architectures inherently balance increased computational demands against enhanced output quality. By decoupling noise estimation from noise suppression into two dedicated neural networks, these systems incur substantial overhead compared to single-stage designs. Empirical studies indicate that isolating the noise-parameter estimation and suppression functions approximately doubles the required floating-point operations: for example, the dual-CNN model DudeNet [120] demands roughly 1.87 GFLOPs vs. 0.94 GFLOPs for the single-stage DnCNN, while delivering comparable denoising efficacy. Similarly, the TSP-RDANet [121] framework employs five residual dynamic attention modules (RDAMs) in its noise-estimation stage and five high-dimensional RDAMs in its suppression stage to achieve a measured compromise between performance and complexity. Memory requirements also escalate: dual-network systems typically possess 25–30% more parameters than their single-stage counterparts [120]. However, advanced network designs can mitigate this burden. For instance, dual-network denoisers like the DCANet [122] and the DCBDNet [123] have been shown to address the issue of increased computational costs and memory consumption growth. The DCANet incorporates a spatial–channel attention module, while the DCBDNet relies on inter-layer skip-connections. While performing on par with state-of the art models (e.g., the DRUNet [124]), these models maintain parameter counts around 1 M—far below 32.6 M in the DRUNet architecture. Their efficiency is further reflected in low computational costs, as of 24.38 GFLOPs for a 256 × 256 grayscale input, and fast inference of 0.183 s on a 1024 × 1024 grayscale image.

Several approaches have been suggested to address explicit noise estimation without obligatory denoising stage. A system based on the DRNE [85] was proposed specifically for noise classification and parameter estimation [125]. For this application, the NN training was augmented with EXIF metadata, categorized into minimal (camera gain, exposure time, sensor temperature) and full metadata sets (additionally including parameters such as dark signal figure of merit, full well capacity, pixel clock rate, sense node gain, sense node reset factor, sensor pixel size, sensor type, and thermal white noise). The noise levels were evaluated across a controlled intensity spectrum, with synthetic noise simulating realistic operating conditions, including low-light and high-gain scenarios. Experimental validation across six datasets demonstrated the method’s effectiveness, with the full metadata model variant achieving RMS errors as low as 0.09 DN for photon-shot noise, 0.35 DN for dark-current shot noise, and 0.47 DN for readout noise on synthetic data, compared to 0.75–1.05 DN RMS errors for the minimal-metadata and metadata-free variants. On real-world noise from a Sony ICX285 CCD and an EV76C661 CMOS sensor, a full metadata model yielded bias and standard deviation below 0.15 DN and 0.45 DN, respectively, for all noise sources, outperforming PGE-Net [109] (bias up to 1.74 DN, Std up to 3.02 DN) and principal component analysis [39] (total-noise RMS of 1.07 DN on synthetic data). In downstream denoising, a full metadata model combined with BM3D [95] achieved PSNRs up to 43.74 dB and SSIM of 0.9839 on real-world scenes, surpassing DRNE [88] + BM3D [95] (43.01 dB/0.9803) and Blind2Unblind [83] (44.10 dB/0.9616) under identical testing conditions (realistic environments, low light, high gain). However, the method’s effectiveness is contingent upon the availability and accuracy of comprehensive camera metadata; performance degrades with consumer-grade cameras that provide limited or inaccurate parameters. Additionally, the model exhibits limitations in scenarios with extreme under- or overexposure and with sensors featuring full well capacities below 24k electrons, due to underrepresented training data. The computational cost remains low at 1.3 ms per inference on a GPU, but the memory requirement for storing extensive metadata may impose practical constraints in resource-constrained environments.

Finally, a conditional denoising transformer (Condformer) was introduced alongside a Locally Noise Prior Estimation (LoNPE) framework for explicit noise-prior embedding [126]. LoNPE estimates Poisson–Gaussian noise parameters from a single raw noisy image. Across the Urban100 dataset [127] with different synthetic Poisson–Gaussian noise levels (α,σ)—ranging from medium (0.05, 0.02) to high (0.2, 0.1)—the LoNPE achieved RMS error of 0.020–0.023 and coefficient of variation below 0.031, outperforming the Makitalo–Foi estimator [128] (RMS 0.041–0.089) with a 300× speedup (0.17 s vs. 56 s per 512 × 512 image). The LoNPE can be simplified and accelerated. A lightweight LoNPE variant runs in 0.01 s with RMS of 0.020–0.025. The Condformer embeds the estimated noise prior into channel-wise self-attention in a Restormer-style [93] U-shaped encoder–decoder with a conditional self-attention module. This results in PSNR and SSIM value increases of 0.34 dB and 0.0018 respectively over state-of-the-art methods (Restormer [93], Uformer [129], MambaIR [130]) on synthetic and realistic benchmarks (Kodak, BSD68 [112], Urban100 [127], SIDD [47]). However, the Condformer’s eight-layer latent module and 27 M parameters incur elevated FLOPs (565 G) and GPU memory (3.8 GB) per 512 × 512 image, leading to 0.37 s inference time—substantially higher than CNN-based denoisers—making it impractical for resource-constrained or real-time applications and scenarios with limited GPU memory.

Table 4 presents the summary of methods discussed in this section.

### 4.4. Source Camera Identification

**Figure 12:**
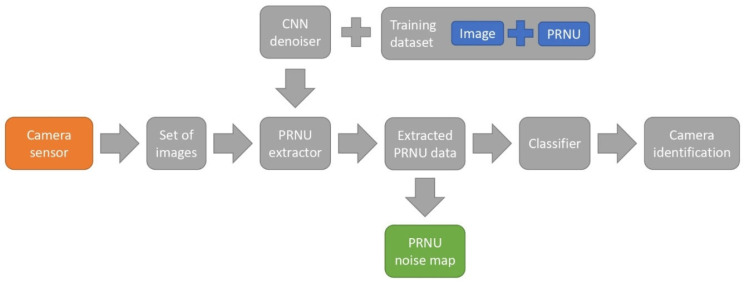
PRNU extraction pipeline for source camera identification.

Digital forensics is an important application for NN-based image processing. In particular, models for determining whether an image was taken on a specific digital camera are being actively developed [131,132,133,134,135,136,137,138,139,140,141,142,143,144,145,146,147]. These methods are usually based on the approximated PRNU(*i*,*j*) values extracted from images with the subsequent classification by camera manufacturers, models or by specific devices [2] (Figure 12). The PRNU extraction approaches have demonstrated high reliability for source camera attribution under mild image processing operations such as moderate JPEG compression and Gaussian blurring, owing to its origin in intrinsic sensor nonuniformities [148,149]. However, PRNU extraction can fail if the high-frequency spatial spectrum is deliberately perturbed by strong denoising filters, geometric desynchronization (e.g., rotation, cropping, scaling), or adversarial noise injection that disrupts spatial correlation. To overcome these limitations, robust fingerprinting schemes can incorporate fusing of the PRNU with complementary low-frequency device-specific fingerprints via deep learning architectures [150]. Nonetheless, PRNU fusing can itself distort the camera noise estimations, deeming such approach unsuitable for noise extraction.

NN methods originally developed for extraction of PRNU(*i*,*j*) or other types of camera fingerprints are of particular interest. In this case, noise suppression isolates the weak but stable high-frequency PRNU component from other low-frequency noise [151]. Therefore, the extracted noise fingerprint can then be used to identify the image source.

In [152], a review of successful applications of CNNs and other machine learning tools for source camera identification is presented. PRNU(*i*,*j*) extraction can be performed using CNNs as well as other methods [153]. One approach to PRNU(*i*,*j*) extraction is the segmentation and suppression of low-frequency noise corresponding to the scene content. Both pre-trained NNs such as the VGG [154] and the specially designed NNs such as the RemNet [155] and the MSFFN [156] can be used to segment and suppress the scene content before the PRNU extraction. Such strategy is used in the Constrained-Net model adapted for video [157], which employs an extended constrained convolutional layer to process color inputs and suppress scene content by learning pixel value prediction errors. The model was evaluated on the VISION dataset [158] with 1539 videos from 28 different camera devices, including 13 instances of the same brand and model to facilitate device-level identification. Videos represented three distinct scenarios (flat, indoor, outdoor) and three versions: native, WhatsApp-compressed, and YouTube-compressed. Under these conditions, the model achieved a peak overall video classification accuracy of 66.5% when aggregating frame classifications via majority vote. Performance varied significantly by scenario, with accuracy rates of 89.1% for the homogeneous ‘flat’ scenario (e.g., walls, blue skies,), 53.7% for ‘indoor’, and 55.2% for ‘outdoor’, indicating a strong dependence on content homogeneity. When evaluating the sensitivity to compression on flat-content videos, the method achieved accuracies of 89.7% on native videos, 93.1% on WhatsApp versions, and 84.5% on YouTube versions. A comparative study demonstrated the critical role of the constrained layer, as its absence significantly decreases the performance. However, this approach necessitates extensive computational resources and training data, requiring over 100,000 frames for training, and its performance degrades considerably with inhomogeneous, dynamic scene content where scene-dependent features dominate. Furthermore, fixed input size requirements and the lack of a patch-based strategy to focus on homogeneous regions within frames limit the flexibility of the approach.

Another group of PRNU(*i*,*j*) extraction methods relies on CNNs to extract features from images. The similarity of the tools suggests that NN denoising can be applied, to some extent, to PRNU(*i*,*j*) extraction. The comparative study [84] shows that common denoising CNNs such as DnCNN [75], ADNet [85], DANet [86], and FFDNet [89] can be adapted for PRNU(*i*,*j*) extraction. For this purpose, the NN was trained using pairs “image-PRNU” with correlation loss function. The study was conducted on the DID [46] dataset using 40 cameras of 11 different models, with images cropped into 128 × 128 and 64 × 64 patches to evaluate the performance under low-resolution conditions. Among the CNN-based methods, the FFDNet achieved the highest average peak-to-correlation energy (PCE) value of 16.5 on 128 × 128 patches, followed by the ADNet (15.1), the DnCNN (13.6), and the DANet (9.6). These results were comparable or superior to non-data-driven methods such as the BM3D [95] (15.8), the DWT [159] (13.5), and the DTCWT [160] (15.3). However, training universal CNN models requires large datasets with at least several thousand images and significant computational resources. For instance, the DANet’s adversarial training is prohibitively time-consuming, and GPU acceleration is often necessary for practical use—the FFDNet required 0.24 s per 1024 × 1024 image on a GPU and 2.05 s on a CPU.

The inclusion of a separate noise-estimation stage in a convolutional neural network is not necessary to achieve effective PRNU extraction. The densely connected hierarchical denoising network DHDN provides an efficient alternative [100]. Its modified U-Net backbone [70], augmented by dense connectivity and residual learning, suppresses scene content and isolates sensor noise. The DHDN was evaluated on two datasets: the DID [46] (74 cameras, 100 images per device) and the DSD [161] (90 devices, 100 images per device) datasets. Cropped images with resolutions of 128 × 128, 256 × 256, and 512 × 512 pixels were used. Performance was measured using kappa statistics and compared with three other methods: the BM3D [95], wavelet denoising [162], and the xDnCNN [163] model. Across the used benchmarks, the DHDN shows improved kappa scores by at least 0.0473 on the DID dataset and 0.0073 on the DSD dataset for all image sizes. The DHDN also demonstrated resilience to JPEG compression at quality factors of 60, 70, 80, and 90. The DHDN, as well as other learning-based approaches, requires substantial computational and memory resources both for training and deployment. Additionally, successful training requires at least 40 images per device used, which rapidly outgrows the requirements for traditional approaches.

Another possible approach to improve the efficiency of camera identification and PRNU extraction is the addition of a residual noise extractor [143]. Specifically, the original image is first denoised using a U-Net [70], then denoised output is subtracted from the original to obtain the residual noise [164]. A ResNet-based convolutional network [67] is then trained on this residual noise to isolate the deterministic PRNU component from a residual noise. The model is able to extract PRNU fingerprints and classify the source camera with 92.41% accuracy on 2194 patches of size 256 × 256 from ten devices in the VISION dataset [158].

Analysis of convolutional neural network training indicates that learning-based PRNU methods impose high memory and computational demands, often requiring tens of gigabytes of GPU memory and hours to days of training on high-end hardware to converge [165]. Likewise, extraction of reference PRNU patterns via maximum-likelihood estimation mandates acquisition and processing of at least 50 flat-field images per device—representing a substantial dataset collection and preprocessing burden that scales linearly with the number of cameras [166]. Furthermore, studies of PRNU robustness under aggressive encoding have shown that heavy image and video compression, such as that employed by social media platforms, reduces PRNU correlation values by over 0.3 relative to the uncompressed material, frequently dropping below reliable detection thresholds and thus degrading identification performance [150].

Table 5 presents the summary of methods discussed in this section.

### 4.5. Other Applications

Apart from noise suppression, identification, characterization, and camera selection, NNs have been used for other applications in noise analysis and image processing. One such application is the NN classification of noise type in images. For instance, a study utilizing a custom CNN architecture achieved a training accuracy of 99.87% and a validation accuracy of 99.92% in classifying facial images corrupted with Gaussian, Poisson, and salt and pepper noise at multiple noise factors (0.05, 0.07, and 0.1), using a dataset of 34,034 preprocessed and augmented images resized to 48x48 pixels [167]. The CNN classifier served as the first stage in an automated pipeline, determining which specialized U-Net denoiser (each trained on a specific noise type) to activate for the subsequent denoising step. This two-stage system demonstrates the utility of noise classification for enabling targeted, noise-specific restoration strategies. Another approach combined deep wavelet scattering transformations with a support vector machine to classify five noise types (gaussian, lognormal, Rayleigh, salt and pepper, speckle). This method, evaluated on a dataset of 1,100 images resized to 28 × 28, achieved a classification accuracy of 91.30% [168]. The noise was applied at a consistent intensity level across all types to ensure a fair comparison; specifically, Gaussian noise was added with a mean of 0 and a variance of 0.01, salt and pepper noise with a noise density of 0.05, speckle noise with a variance of 0.05, while lognormal and Rayleigh noise parameters were scaled to produce a similar visual degradation and signal-to-noise ratio. In [169], a combination of a traditional filtration algorithm and a NN was proposed for noise estimation and classification. This method applies discrete wavelet transformation for high frequency band extraction followed by the noise level estimation. However, this approach does not provide correspondence of digital estimates to physically reasonable noise parameters. In addition, reported precision and recall of 1.0 raises suspicions of overfitting the model; additional analysis of model performance was not reported.

Based on the PRNU extraction methods and JPEG error level analysis (ELA), methods for artificial image detection have been developed [170]. Testing was conducted using a balanced dataset of 918 images (459 AI-generated, 459 real), extracting PRNU and ELA features from central 512 × 512 crops for input to a custom CNN. The PRNU-based model achieved 0.95 accuracy and 0.95 F1-score, while the ELA-based model reached 0.98 accuracy and 0.98 F1-score on the validation set. Similar results were achieved with the Siamese NN for deepfake image detection [171]. Testing was conducted on five benchmark deepfake datasets (FF++, Celeb-DF, DFD, DeeperForensics, DFDC) using a Siamese two-stream network with the Inception-v3 backbone; video-level predictions were aggregated over 25 frames. The proposed SiamNet achieved frame-level accuracies of 98.7% on FF++, 94.2% on Celeb-DF, 92.1% on DFD, 99.8% on DeeperForensics, and 86.9% on DFDC, and video-level accuracies of 99.9% on FF++, 98.3% on Celeb-DF, 96.08% on DFD, 100% on DeeperForensics, and 89.2% on DFDC.

## 5. Discussion

Current solutions such as the EMVA 1288 standard provide accurate estimation of noise parameters, but they are complex and computationally intensive. Qualitative estimation methods based on automatic scene segmentation [13,24,25,26] use only 2–4 images, but require specialized setups. Simplified statistical methods for noise estimation and suppression using Gaussian [14] or Poisson [17] models, or their combinations [15,16], work using only a single image, but are not as reliable and depend on specific shooting conditions and camera parameters.

Compared to conventional approaches NNs offer several advantages. For example, they are able to account for complex dependencies between noise and signal, thereby greatly increasing the flexibility and versatility of NN models. The main methods and their features are shown in Table 6.

In recent years, CNN-based autoencoders, transformers, and generative models [55,56,57,58,59,60,61,70,73,74,75,81,84,85,86,87,88,89,90,92,93,94,96,97,98,99,100,101,102,103,104,105,106,107,108,109,115,116,117,120,121,122,123,124,125,126,129,130,131,132,133,134,135,136,137,138,139,140,141,142,143,144,145,146,147,154,155,156,157,164,167,168,169,170,171] have demonstrated promising results for noise estimation and suppression. CNNs generally demonstrate high accuracy of noise feature extraction with unsupervised training. Trained NNs often require only a single image to accurately estimate individual noise components. In this aspect, NN-based approaches significantly outperform conventional methods, which often require hundreds of images [8]. NN models are also several times faster than conventional methods based on automatic scene segmentation [13,24,25,26] and provide more accurate estimations than single-image methods [14,15,16,17].

The flexibility of NN architectures and approaches facilitates the adaptation of NN-based methods for a wide variety of applications in camera and image noise processing [1]. For example, NN-based noise suppression methods proved useful in both direct estimation of several noise characteristics [70,75,84,85,86,88,89,90,94,96,97,98,99,100,101,102,103,104,105,108,109,117,122,126] and in mapping the approximated pixel spatial heterogeneity for source camera identification [70,75,84,86,88,90,125]. GAN models show promise for noise modeling and generation [56,57,58,59,60,61], suppression [81,94], and extraction [81]. Last, but not least, some transformer architectures show exceptional ability for noise parameter evaluation [90,126].

The ability to generalize and integrate additional context data is a significant advantage of NNs over traditional approaches. For example, integration of EXIF metadata and information about shooting conditions into the training process improved the quality of noise suppression and camera identification [125].

However, for all their advantages, NN-based methods also have their limitations that manifest across multiple dimensions of computational efficiency and practical deployment.

**Training Dataset and Development Constraints**

NN training fundamentally limits the development speed due to the requirements for high-quality, domain-specific datasets that are notoriously difficult to create. The acquisition of hundreds or thousands of real-world images for training and testing demands significant time and labor investment, exemplified by datasets like SIDD [47] requiring ~30,000 raw–RGB image pairs from multiple camera models under diverse conditions. The computational burden extends beyond data collection: training modern architectures like the Condformer [126] (27M parameters, 565 GFLOPs) or CANGAN [57] frameworks can require days to weeks on high-end GPU clusters, representing substantial infrastructure costs that traditional methods avoid entirely.

**Computational Efficiency Trade-offs**

The computational landscape reveals stark disparities between neural network-based and traditional approaches. While traditional methods like the BM3D [95] maintain consistent, moderate resource requirements (71 MB memory for 512 × 512 images, ~0.2 GFLOPs), learning-based methods exhibit dramatic variability. Efficient architectures like the NoiseFlow [56] achieve competitive performance with fewer than 2500 parameters, while resource-intensive models like the FADNet [90] demand 22 M parameters and ~150 GFLOPs per forward pass. This represents a three-order-of-magnitude difference in parameter count within the neural network domain alone.

Dual-network architectures embody the performance–efficiency trade-off: methods like CBDNet [105] and NERNet [108] that separate noise estimation from suppression approximately double computational overhead compared to single-stage designs (DudeNet [120]: 1.87 GFLOPs vs. DnCNN [75]: 0.94 GFLOPs), while delivering only marginal PSNR improvements (typically 0.5–2 dB). However, specialized designs like the FBI-Denoiser [109] demonstrate that architectural innovation can achieve substantial time-efficiency gains.

**Memory and Deployment Scalability**

Memory requirements present another critical constraint. Contemporary neural denoisers typically consume 2–4 GB of GPU memory during inference, compared to traditional methods that require less than 100 MB. This disparity becomes prohibitive for deployment on resource-constrained devices or real-time applications. The asymmetric resource utilization—computationally intensive training followed by relatively lightweight deployment—creates infrastructure challenges distinct from traditional methods’ consistent moderate load throughout their pipeline.

**Noise Separation and Interpretability Challenges**

Qualitative separation of scene-dependent noise from photosensor noise remains a fundamental limitation. For valid comparison with traditional camera characterization methods, NN-based pipelines must distinguish between these types of noise within their processing workflows. Currently, this separation is typically achieved only partially for individual noise components (e.g., PRNU extraction), but comprehensive simultaneous separation of all four EMVA 1288 [8] noise types remain unresolved. The interpretability issue compounds this challenge: features extracted from architectures adapted for noise processing cannot be reliably classified as photosensor vs. image noise, undermining their utility for camera characterization applications.

**Benchmarking and Evaluation Gaps**

Evaluation and comparison of traditional and learning-based methods are complicated by fundamental differences in performance. This primarily applies to estimates of computational complexity, memory usage, and the efficiency of the methods used in relation to human labor when using different methods. Traditional methods offer deterministic, physics-based parameters estimation suitable for standardized camera characterization, but they tend to be inefficient in terms of human labor. Learning-based methods excel at statistical pattern recognition optimized for specific datasets, offering labor-efficient usage after deployment at the cost of computational complexity and strict requirement for training datasets. This methodological divergence necessitates development of hybrid evaluation frameworks that can assess both accuracy and computational efficiency across different paradigms.

**Research Priorities and Future Directions**

Considering these limitations, several critical research priorities emerge:

- Physics-informed architecture design: Developing neural networks that explicitly model the four-component noise structure defined by EMVA 1288 [8], enabling direct separation of photosensor noise from scene artifacts while maintaining computational efficiency.
- Standardized synthetic dataset creation: Establishing large-scale, validated synthetic datasets that accurately model complex noise pipelines of modern smartphones and cameras, reducing dependency on labor-intensive real-world data collection.
- Efficiency-optimized architectures: Investigating architectural innovations that achieve the accuracy of dual-network models while approaching single-network computational costs, particularly for real-time and mobile deployment scenarios.
- Hybrid evaluation frameworks: Creating benchmarking protocols that quantitatively compare learning-based and traditional methods across multiple dimensions—accuracy, computational cost, memory efficiency, and physical parameter interpretability.
- Interpretable feature extraction: Developing methods to ensure that neural network outputs correspond to physically meaningful noise parameters, enabling their use in camera characterization and forensic applications where interpretability is paramount.
- Adaptive computational scaling: Designing networks capable of trading accuracy for efficiency based on deployment constraints, allowing the same architecture to serve both high-accuracy offline applications and resource-constrained real-time scenarios.

## 6. Conclusions

Different approaches to neural network-based digital camera and image noise estimation, suppression, classification, extraction, etc., are being actively developed. These methods can be applied for image quality enhancement, digital forensics, device classification, comparison, identification, etc. Modern neural network architectures can account for both the physical properties of noise and the contextual data, such as shooting parameters. This additional information increases the accuracy and reliability of noise estimation and classification.

The convolutional neural networks discussed in this paper demonstrate high accuracy extraction of real-world noise features, while generative models demonstrate efficient generation of synthetic noise. Special attention needs to be paid to the possibility of using neural networks for extraction, interpretation, and separation of noise components arising from image or photosensor noise.

Neural network-based methods for image processing and camera noise estimation demonstrate great potential for further improvement and practical application in different areas, such as image restoration, signal-to-noise ratio enhancement, forensics, and image source identification.

## Figures and Tables

**Table 1:** Noise and technical parameters of several digital cameras.

Parameters	Canon EOS M100	PixeLink PL-B781F	Retiga R6
Pixel size, μm	3.7	3.5	4.6
Full resolution, MP	24	6.6	5.9
Sensor	CMOS	CMOS	CCD
Type	Consumer	Machine vision	Microscopy
Bit depth, bit	13.5	10	14
σ*_dt_*, DN	2.479 ± 0.004	0.351 ± 0.004	4.46 ± 0.01
*K*, DN/e	0.781 ± 0.006	0.093 ± 0.005	0.84 ± 0.03
DSNU, DN	0.191 ± 0.005	0.66 ± 0.02	0.50 ± 0.01
PRNU, relative units	0.0092 ± 0.0003	0.0075 ± 0.0002	0.0033 ± 0.0001

**Table 2:** Noise synthesis and modeling neural network architectures comparison.

Architecture	Numerical Results	Training Conditions and Datasets	Notes
NoiseFlow [56]	NLL: −3.521 nats/pixel KL: 0.008	SIDD [47] with ~30,000 raw–RGB image pairs, 5 smartphone cameras, ISO 50–10,000	Conditional normalizing flow for complex signal-dependent noise modeling, <2500 parameters
CANGAN [57]	KL: 0.00159, DnCNN [75]: PSNR: 48.71 dB, SSIM: 0.992	SIDD [47], ~24,000 raw–RGB image pairs	U-Net [70] based noise generator + camera encoding network for camera-specific noise
ResNet-based frameworks [67,74]	KL: 0.0211 DnCNN [75]/U-Net [70]: PSNR: 50.13/51.40 dB, SSIM: 0.9891/–	SIDD [47], SID [49] Canon EOS 5D4, Nikon D850, Sony RX100VI, HUAWEI P40 Pro	Contrastive learning for fine-grain noise parameter estimation with 4-tuple model
C2N [59]	KL: 0.1638 DnCNN [75]/DIDN [79]: PSNR: 33.76/35.35 dB, SSIM: 0.901/0.937	SIDD [47], DND [48], unpaired clean and noisy images	Unsupervised GAN for noise modeling without paired data
Noise2NoiseFlow [60]	NLL: −3.501 nats/dim, KL: 0.0265, DnCNN [75]: PSNR: 52.80 dB, SSIM: 0.984	SIDD [47], ~500,000 patches 32 × 32, ISO 100–3200	Combines Noise2Noise [61] with normalizing flow, eliminates need for clean ground truth
DCD-Net [81]	PSNR: up to 51.40 dB, SSIM: up to 0.992	Kodak, BSD300 [51], Set14 [82], SIDD [47] raw–RGB validation	Iterative training «denoise-corrupt-denoise» on noisy images only, denoising enhancement

**Table 3:** Denoising neural network architecture performance comparison.

Architecture	Numerical Results	Training Conditions and Datasets	Notes
DRNE [88]	FFDNet [89]: PSNR: 33.68 dB Average error (noise estimation): up to 0.32 dB	Kodak, McMaster [52], BSD500 [53], synthetic Gaussian noise	16-layer CNN for pixelwise noise variance mapping, signal-dependent noise estimation
FADNet [90]	PSNR: 41.36 dB	Nam [54], SIDD [47], 1200 random 512 × 512 patches	Frequency–domain attention mechanism with encoder–decoder, 22 M parameters, ~150 GFLOPs
GAN-based denoiser [94]	PSNR: 39.29 dB, SSIM: 0.915	SIDD [47]	Three-component: generator + dual-path U-Net [70] denoiser + discriminator, 15.6 M parameters, 68.9 GFLOPs

**Table 4:** Neural network architectures for noise estimation and removal.

Architecture	Numerical Results	Training Conditions and Datasets	Notes
CBDNet [105]	PSNR: up to 41.31 dB, SSIM: 0.9421	DND [48], Nam [54] dataset, synthetic + real-world	Two-subnetwork: 5-layer noise estimation + 16-layer U-Net [70] denoising
NERNet [108]	PSNR: up to 40.10 dB, SSIM: 0.942	SIDD [47], Nam [54], BSD68 [112]	Enhanced CBDNet [105] with pyramid feature fusion and attention mechanisms
FBI-Denoiser [109]	PSNR: up to 48.02 dB, SSIM: up to 0.9797 1560× speedup in estimation	BSD68 [112], FiveK [113], FMD [114], SIDD [47], DND [48]	GAT [110] preprocessing, 0.21 s inference time, 340 K parameters
PCANet + ResNet101 [117]	Mean estimation error: 0.22, patch selection accuracy: 92%	100 images of BSD300 [51], 1M+ training patches	Global statistical noise estimation with chi-square distribution
Metadata-enhanced model [125]	RMS errors: from 0.09 to 0.47 DN, PSNR: up to 43.74 dB	Sony ICX285 CCD, EV76C661 CMOS, synthetic data	DRNE-based [88] with EXIF metadata integration, 1.3 ms inference time
Condformer + LoNPE [126]	RMS error: up to 0.023, 300× speedup, PSNR improvement: 0.34 dB	Urban100 [127] with synthetic Poisson-Gaussian noise	Transformer-based with noise prior embedding, 27 M parameters, 565 GFLOPs

**Table 5:** Source camera identification neural network method comparison.

Architecture	Numerical Results	Training Conditions and Datasets	Notes
Constrained-Net [157]	Video classification accuracy: 66.5% overall, 89.1% flat scenes	VISION [158], 1539 videos, 28 camera devices, >100 K training frames	Extended constrained convolutional layer for video PRNU extraction
CNN adaptations [84]	PCE: up to 16.5 (FFDNet [89])	DID [46], 40 cameras, 11 models, 128 × 128 and 64 × 64 patches	Adaptation of denoising CNNs for PRNU extraction with correlation loss
DHDN [100]	Kappa improvement: at least 0.0473	DID [46] (74 cameras), DSD [161] (90 devices), 100 images per device	Modified U-Net [70] with dense connectivity for sensor noise isolation
ResNet-based extractor [143]	Classification accuracy: 92.41%	VISION [158], 2194 patches 256 × 256, 10 devices	U-Net [70] denoising with ResNet [67] residual noise extraction for PRNU fingerprints

**Table 6:** Features and description of main neural network-based methods.

Architecture	Features	Description
NOISE SUPPRESSION		
DnCNN [75]	Unsupervised noise suppression	Residual convolutional neural network that uses unsupervised training to suppress noise in images
DRNE [88]	Pixel-based noise mapping	Network for noise estimation capable of utilizing metadata and additional information
CBDNet [105], NERNet [108], FBI-Denoiser [109], DCANet [122], DCBDNet [123]	Two-network structure: noise estimator and noise suppressor	Separation of noise estimation and suppression allows them to be improved separately
Other neural networks for noise suppression [94,96,97,98,99,100,101,102,103,104]	Convolutional (U-Net based, etc.) or generative-adversarial networks	Can be used for approximated PRNU extraction
**SOURCE CAMERA IDENTIFICATION**		
U-Net modifications [70]	Feature extraction	U-Net’s high spatial resolution feature extraction capability is adapted for approximated PRNU extraction
DnCNN [75], FFDNet [89], ADNet [85], DANet [86]	Adapted noise suppression networks	Can be adapted for approximated PRNU extraction
Constrained-Net [157]	Works with video data	Requires large (over 100 thousand frames) amount of video data for training
**SYNTHETIC DATASET GENERATION**		
NoiseFlow [56], CANGAN [57], C2N [59]	Noise synthesizers	Models based on generative-adversarial networks or normalizing flows
Noise2NoiseFlow [60], DCD-Net [81]	Noise suppression with a noise synthesizer	Improving noise suppression by utilizing a noise synthesizer or by using only noisy data for training

## Data Availability

Data sharing is not applicable.

## Abbreviations

The following abbreviations are used in this manuscript:

AIRM	Adaptive Instance Residual Module
AWGN	Additive White Gaussian Noise
BM3D	Block-Matching and 3D Filtering
CNN	Convolutional Neural Network
CPU	Central Processing Unit
DN	Digital Number
DS	Dilation Selective (block)
DSNU	Dark Signal Non-Uniformity
DWT	Discrete Wavelet Transformation
EMVA	European Machine Vision Association
GAN	Generative Adversarial Network
GAT	Generalized Anscombe Transformation
GPU	Graphics Processing Unit
KL	Kullback–Leibler (Divergence)
NLF	Noise Level Functions
NLL	Negative Log-Likelihood
NN	Neural Network
PCE	Peak-to-Correlation Energy
RDAM	Residual Dynamic Attention Modules
ReLU	Rectified Linear Unit
RMS	Root Mean Square (error)
PRNU	Photo Response Non-Uniformity
PSNR	Peak Signal-to-Noise Ratio
SSIM	Structural Similarity Index Measure
Std	Standard deviation
